# An approach for monitoring temperature on fruit surface by means of thermal point cloud

**DOI:** 10.1016/j.mex.2022.101712

**Published:** 2022-04-26

**Authors:** Nikos Tsoulias, Sven Jörissen, Andreas Nüchter

**Affiliations:** a Department Horticultural Engineering, Leibniz Institute for Agricultural Engineering and Bioeconomy (ATB), Max-Eyth-Allee, Potsdam 14469, Germany; b Department of Informatics VII—Robotics and Telematics, Julius-Maximilians-University Würzburg, Würzburg 97074, Germany

**Keywords:** Point cloud, Thermal point cloud, Fruit temperature, Sunburn, Food quality, Precision Horticulture

## Abstract

Heat and excessive solar radiation can produce abiotic stresses during apple maturation, resulting fruit quality. Therefore, the monitoring of temperature on fruit surface (FST) over the growing period can allow to identify thresholds, above of which several physiological disorders such as sunburn may occur in apple.

The current approaches neglect spatial variation of FST and have reduced repeatability, resulting in unreliable predictions. In this study, LiDAR laser scanning and thermal imaging were employed to detect the temperature on fruit surface by means of 3D point cloud. A process for calibrating the two sensors based on an active board target and producing a 3D thermal point cloud was suggested. After calibration, the sensor system was utilised to scan the fruit trees, while temperature values assigned in the corresponding 3D point cloud were based on the extrinsic calibration. Whereas a fruit detection algorithm was performed to segment the FST from each apple.

• The approach allows the calibration of LiDAR laser scanner with thermal camera in order to produce a 3D thermal point cloud.
• The method can be applied in apple trees for segmenting FST in 3D. Whereas the approach can be utilised to predict several physiological disorders including sunburn on fruit surface.

## Graphical abstract

**Figure:**
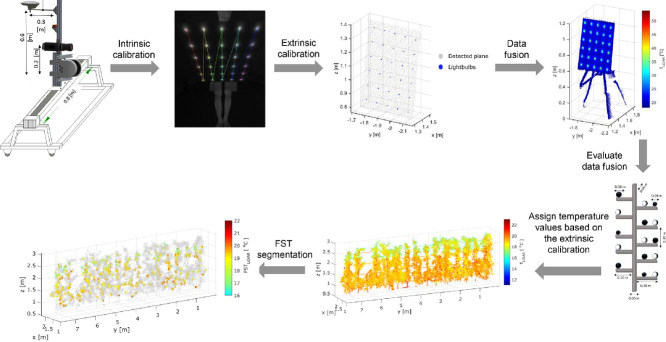

Specifications table

**Table:** 

Subject Area:	Agricultural and Biological Sciences
More specific subject area:	Horticultural Technology 3D point cloud analysis Machine vision
Method name:	An approach for monitoring fruit temperature in 3D
Name and reference of original method:	The computational methods are inspired by the literature and primarily: • Borrmann, D., Nüchter, A., Dakulović, M., Maurović, I., Petrović, I., Osmanković, D., & Velagić, J. (2014). A mobile robot based system for fully automated thermal 3D mapping. *Advanced Engineering Informatics, 28*(4), 425–440. 10.1016/J.AEI.2014.06.002 • Tsoulias, Nikos, Dimitrios S. Paraforos, George Xanthopoulos, and Manuela Zude-Sasse. "Apple shape detection based on geometric and radiometric features using a LiDAR laser scanner." Remote Sensing 12, no. 15 (2020): 2481. 10.3390/rs12152481
Resource availability:	Python code (www.python.org) was written and a Python code script file has been created.

## Introduction

Excess solar radiation, elevated temperatures and low relative humidity are the main cause of abiotic stress on the fruit skin in orchards. In apples (*Malus* x *domestica* Borkh.), such field conditions suppress anthocyanin accumulation resulting in low pigmentation on fruit surface [42]. Enhanced temperatures on apple surface postpones starch degradation and consequently the conversion into sugars, especially in high altitudes [32]. Similar conditions can enhance respiration rate or reduce net photosynthesis, which in turn influences fruit growth rate at cell division stage [6,26], producing apples of smaller size [31]. Whereas, after fruit set, high temperatures (35°C) have also been related with decreased firmness levels at harvest stage [40].

Several physiological disorders can occur in the exposed surface of apples including sunburn, compromising fruit quality, storability and enhancing food waste. Longer periods of solar radiation and high temperatures are susceptible to appear, over the growing season of apples, due to climate change increasing yield losses. Recent reports mentioned annual yield losses up to 10 % in the US and New Zealand [22,33], 40 % in Australia [20], and from 10 to 50 % in south Africa [29], in apple orchards. Fruit skin temperature (FST) can be utilised as a reliable indicator to identify types of sunburn symptoms in apples [23]. Schrader et al. [24] found that when FST reaches around 52°C for longer than 10 min the epidermal cells exposed directly to the sun die, destroying the photosynthetic mechanism of the fruit. The combination of high ultraviolet radiation and FST (46-49°C) results in browning sunburn, discoloring the exposed peel due to chlorophyll degradation, and producing different levels of bronzing in the flesh [21]. Furthermore, shaded fruit skin suddenly exposed to moderate temperatures (< 31 °C) may result in photooxidative stress [9]. However, the surface Incidence and severity of the damage depend on a complex interplay of these factors together with the biochemical, physiological, and morphological condition of the apple, all of which are a function of the phenological stage, cultivar and adaptation to meteorological conditions. The monitoring of FST over the season structural characteristics in apple trees provides decisive knowledge for management within the orchard.

Contact methods for measuring FST include pushing the sensory bulb of a thermometer under the peel of the apple and inserting thermocouples on the fruit surface. However, the latter techniques wound the fruit surface, decreasing the repetitiveness of the measurement [18]. Whereas, the spatial variation of temperature on the fruit surface is neglected since the measurement takes place at one location. However, non-destructive techniques for measuring the FST have gradually gained a substantial advancement by the implementation of numerous two-dimensional machine vision systems in agriculture, using color or spectral information combined with thermal imaging. However, fruit localization and segmentation, for the monitoring of FST, based only on thermal information may be biased or fail due to similar temperature [11]. Chandel et al., [8] coupled an RGB camera with a thermal module to model FST, revealing a coefficient of determination (R^2^) up to 0.90 with the FST derived from the micro-climate sensor. A similar system was developed using colour information to obtain apple size and infra-red images for estimating FST in real time [25]. However, fruit segmentation based on captured images are susceptible to light variations and may be biased due to equal colour or shading condition of fruit, leaves and woody parts [17,37].

Three-dimensional (3D) vision systems received attention in horticulture, allowing to overcome the limitations of 2D imaging methods [14]. Hence, the shape of apple trees can be described using high resolution 3D point cloud data that can be acquired either directly from cameras such as RGB-depth [10] or using photogrammetric techniques with spectral [5,16] and RGB cameras [12,15]. However, also in the 3D analysis, the variation of light shading conditions within the canopy reduces the quality of the 3D point cloud acquired from camera systems [30]. The 3D point cloud and radiometric data can be generated from light detection and ranging (LiDAR) sensors, which operate based on time-of-flight principle (ToF). An active laser diode emits a laser beam and reflected photons describe the object surface, overcoming the effects of varying light conditions. The sensor is typically mounted on terrestrial platforms and operates parallel to the tree rows scanning the canopy surfaces from both sides, analysing each tree even in large-sized areas such as landscape and forests. Several studies in fruit production used LiDAR data to monitor tree geometry such as the canopy volume over tree growing period [7,41]. By means of this data, the spatio-temporal development of leaf area and woody parts were monitored trees within two seasons, utilising geometric and radiometric features [39]. Whereas, fruit detection approaches were proposed for segmenting shape and size in apples [13]. Despite the potential of 3D data in plant phenotyping, information on tree or fruit temperature is not present by default, while methods have been proposed and investigated in the field of architecture [3,19] and robotics [1,4]. Recently, a terrestrial LiDAR laser scanner was coupled with a thermal camera for reconstructing the 3D thermal point cloud in avocados [34]. Individual trees scanned from west and east side to acquire leaf temperature, revealing an ± 5 °C mean bias error compared with manual readings from both sides due to asynchronicity of LiDAR data with camera pixels. However, the scope of the conducted studies has been limited to small-scale validation concept. No study has been reported yet to observe the suitability of thermal - LiDAR 3D sensing for estimating FST in field conditions, an essential step for modeling FST and improve sunburn management strategies.

The present study aimed to (i) develop a robust method for merging 3D LiDAR data with thermal images, (ii) evaluate the data fusion under laboratory and field conditions using a metal tree target and (iii) the segmentation of apple fruit surface temperature from 3D thermal tree point clouds.

## Materials and methods

### Site description

The experiment was conducted in the Leibniz Institute of Agricultural Engineering and Bioeconomy (ATB) experimental station located in Marquardt, Germany (Latitude: 52.466274° N, Longitude: 12.57291° E). The orchard is located on an 8% slope with southeast orientation. The orchard is planted with trees of Malus  ×  domestica Borkh. ‘Gala’ and ‘JonaPrince’, and pollinator trees ‘Red Idared’ each on M9 rootstock with 0.95 m distance between trees, trained as slender spindle, which form the majority of apple trees in world-wide production, with an average tree height of 2.5 m. Trees are supported by horizontally parallel wires.

### Instrumentation

**Fig. 1:**
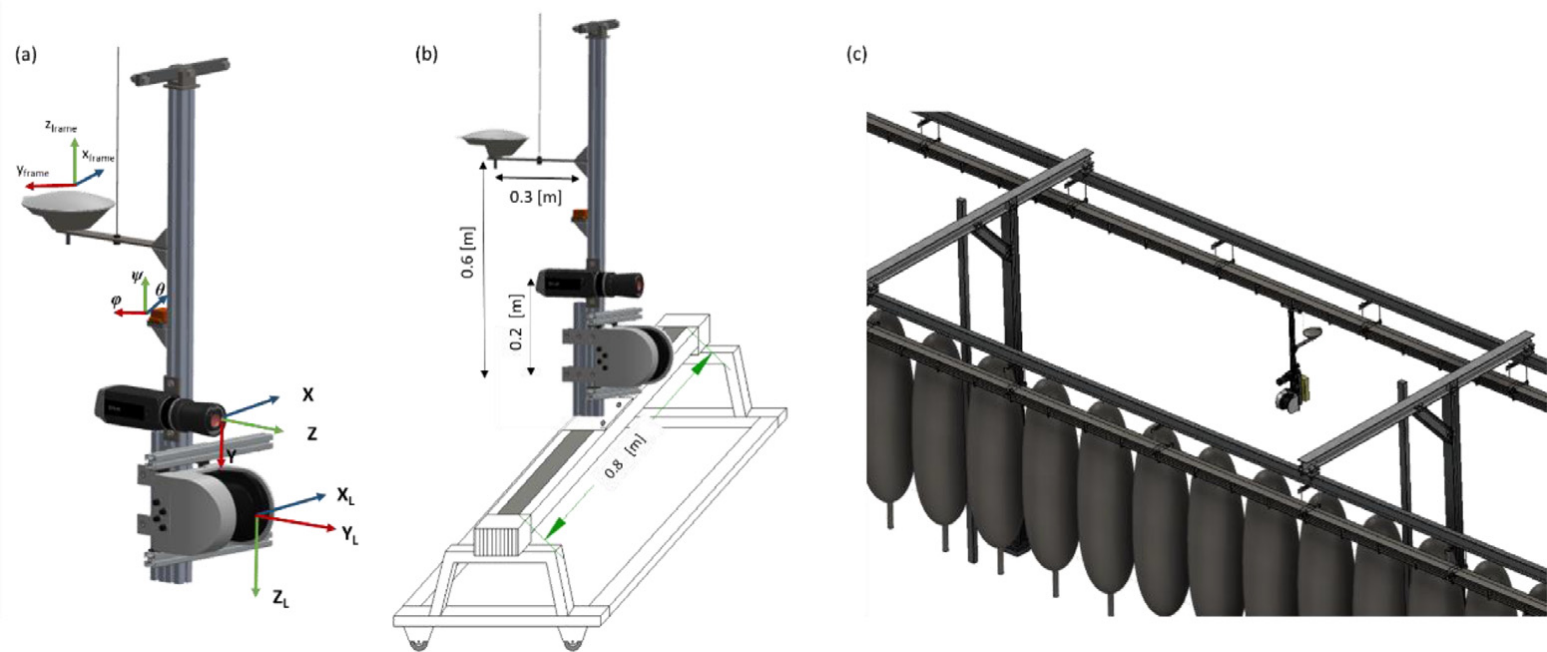
Representation of (a) the sensor-frame system; mounted on (b) linear tooth-belt and (c) circular conveyor system.

A rigid aluminium frame carrying the sensors (Fig. 1a) is mounted on a rigid linear tooth-belt conveyor system (Module 115/42, IEF Werner, Germany) of 0.8 m length, employing a servo positioning controller (LV-servoTEC S2, IEF Werner, Germany) (Fig. 1b), to perform intrinsic and extrinsic calibration using an active pattern with clearly defined heat sources (*m* = 30). The linear conveyor moved at 20 mm s^−1^ (± 0.05 mm accuracy) forward speed. A mobile 2D LiDAR sensor (LMS-511, Sick AG, Waldkirch, Germany) was mounted vertically on the metal frame at 0.7 m above the ground level. The LiDAR sensor configured with a 0.1667° angular resolution, 25 Hz scanning frequency, a scanning angle of 180 and a wavelength of 905 nm. Additionally, a thermal camera (A655sc, FLIR Systems Inc., MA, USA) placed at 0.2 m distance above the laser scanner. The camera has a spatial resolution of 640 × 480 pixels at 50 Hz, with a spectral range from 7.5 to14 μm, an operational temperature range from −40°C to 150°C and a thermal resolution < 0.05°C. A lens (T198065, FLIR Systems Inc., MA, USA) with a focal length of 6.5 mm (diagonal 80^o^) is attached to the camera. The calibration was carried out in a room with no windows, controlled ventilation and temperature (15^°^C). The LiDAR and the thermal camera were connected via Ethernet to a laptop with software developed in LabVIEW (version NXG 5.1, National Instruments, Texas, USA) for data acquisition. The positioning controller of the linear conveyor connected to the same computer with a RS-232 serial port using the S2 Commander software (version 4.1.4201.1.1, IEF Werner, Germany) for configuration and operation.

After calibration, the phenotype sensing system was mounted on a circular conveyor platform, established in the experimental apple orchard (TechGarden), employing an electrical engine working with 50 Hz (DRN71, SEW Eurodrive, Germany) and stainless-steel chain with mechanical suspensions for varying plant sensors (Fig 1c). A real time kinematic global navigation satellite system (AgGPS 542, Trimble, Sunnyvale, CA, USA) is used to geo-reference the data and an inertial measurement unit (MTi-G-710, XSENS, Enschede, Netherlands) to acquire orientation information are arranged on the sensor frame. The root mean square error (RMSE) of orientation noted at 0.25° for roll (*φ*), pitch (*θ*) and yaw (*ψ*). Furthermore, an RTK-GNSS (AgGPS 542, Trimble, Sunnyvale, CA, USA) used for georeferencing each individual scanning profile of the 3D point cloud. The horizontal and vertical accuracy of the RTK-GNSS is ± 25 mm + 2 ppm and ± 37 mm + 2 ppm, respectively. The IMU is placed 0.3 m aside from the LiDAR sensor, while the receiver antenna of RTK-GNSS is mounted 0.6 m abive the laser scanner (Fig. 1). This phenotyping platform was established in a single row of the experimental apple orchard in 2020. The platform enables the automated monitoring of 109 trees in one row of 84 m length.

### Data pre-processing

The accurate projection of thermal information on the 3D point cloud requires calibration of the sensor frame system. More specifically, the process consists of two parts: (i) the intrinsic calibration of the thermal camera for determining camera matrix and distortion parameters, and (ii) the extrinsic calibration between camera and LiDAR coordinate system to define rotation and translation. The calibration tool chain is written in Python 3.8 (Python Software Foundation) and uses the OpenCV library (Bradski & Kaehler, 2008) for image processing and Open3D [36] for point cloud processing. The following sections describe the methodology behind the calibration tool chain.

### Intrinsic calibration

**Fig. 2:**
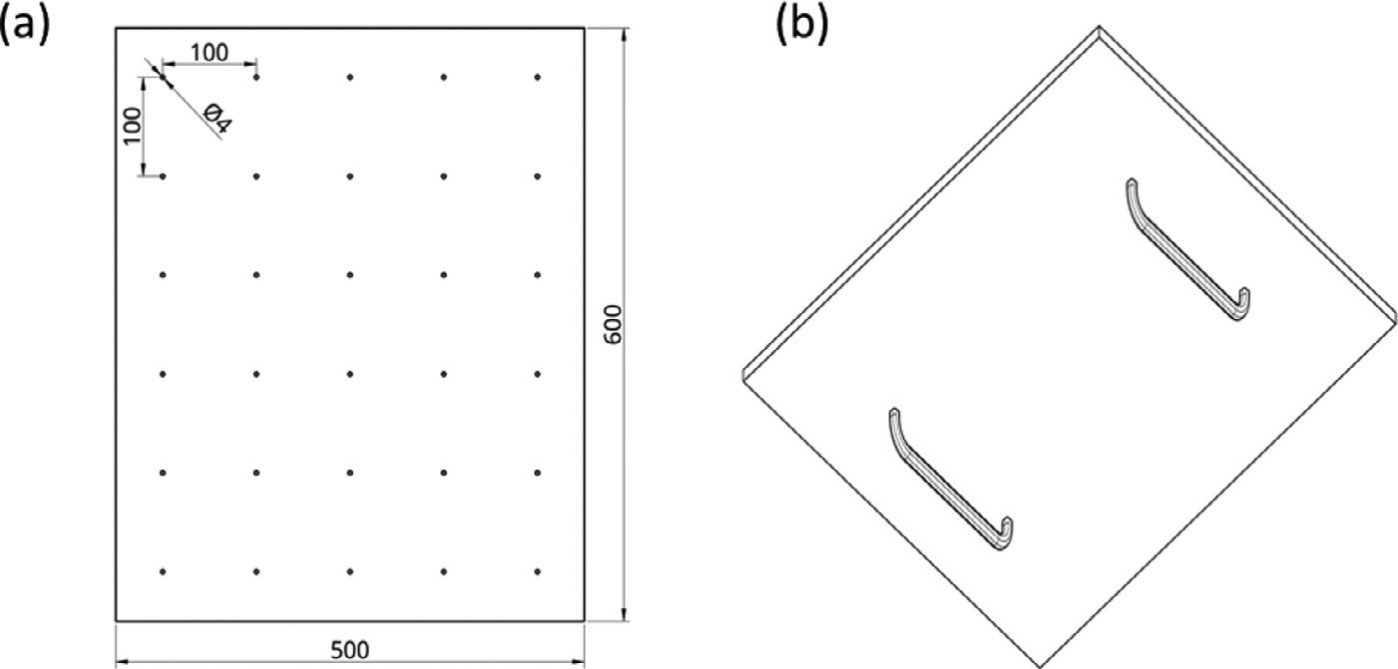
Lightbulb blob calibration pattern, (a) front view with dimensions and (b) back view with handles.

The intrinsic calibration of a thermal camera is typically estimated by detecting geometric features from patterns in the image, such as corners from chessboard patterns of known dimensions. However, chessboard patterns are not suitable for calibrating thermal cameras, since even when passively heated, the temperature difference between black and white areas is not sufficient to be identified as distinct corners. Therefore, as suggested in [2], an actively heated lightbulb pattern was constructed (Fig. 2 a,b). The pattern consisted of a wooden board (500 × 600 mm), containing 30 (*m*) 12V lightbulbs of 4 mm diameter, arranged in a 5 × 6 grid with a distance of 100 mm.

The lightblubs appeared as blobs in thermal images, therefore, the OpenCVs *SimpleBlobDetector* (Bradski, G., & Kaehler, A. 2000) algorithm was used to obtain their respectivecoordinates. The latter was based on the region of connected points, which determined by colour thresholding, grouping and size of detected blobs. More specifically, the source images were converted to several binary images, thresholding was applied, starting with *minThreshold*, ending with *maxThreshold* in *tresholdStep* increments. The segmented white pixels were grouped and, their overall shape and size is estimated, while the grouped pixels across all thresholded images were combined for calculating the center and radius for each individual blob. Moreover, the size of the blobs (*minArea, maxArea*) was configured. Three sets of parameters were empirically determined and are further noted as sensitivity (*s)*.

The intrinsic parameters of the thermal camera, namely the focal length in x) and y (f||y) direction, the camera center $\left(c_{x},c_{y}\right)$ as well as radial ($k_{1},k_{2},k_{3}$ and tangential ($p_{1},p_{2}$) distortion were determined using [35]. The relationship of a 3D real world point $O={\left(X,Y,Z\right)}^{T}$ and its 2D projection $o={\left(x,y\right)}^{T}$ on the sensor plane is defined by

(1) $$\left(\begin{array}{c} x\\ y \end{array}\right)=\left(\begin{array}{c} f_{x}X+Zc_{x}\\ f_{y}Y+Zc_{y} \end{array}\right)$$

Expressing (Eq. (1)) with homogeneous coordinates yields

(2) $$\left(\begin{array}{c} x\\ y\\ 1 \end{array}\right)=\left[\begin{array}{ccc} f_{x} & 0 & c_{x}\\ 0 & f_{y} & c_{y}\\ 0 & 0 & 1 \end{array}\right]\left(\begin{array}{c} X\\ Y\\ Z \end{array}\right)$$

with

(3) $$A=\left[\begin{array}{ccc} f_{x} & 0 & c_{x}\\ 0 & f_{y} & c_{y}\\ 0 & 0 & 1 \end{array}\right]$$

as the camera matrix. The distorted image points ${\left(x_{d},y_{d}\right)}^{T}$ are calculated as

(4) $$\left(\begin{array}{c} x_{d}\\ y_{d} \end{array}\right)=\left(\begin{array}{c} x\left(1+k_{1}r^{2}+k_{2}r^{4}+k_{3}r^{6}\right)+2p_{1}xy+p_{2}\left(r^{2}+2x^{2}\right)\\ y\left(1+k_{1}r^{2}+k_{2}r^{4}+k_{3}r^{6}\right)+p_{1}\left(r^{2}+2y^{2}\right)+2p_{2}xy \end{array}\right)$$

with $r=\sqrt{x^{2}+y^{2}}$.

For a calibration pattern of known dimensions with a set of m lightbulb features, n images were captured and the reprojection error of the detected features minimised as

(5) $$\sum_{i=1}^{n}\sum_{j=1}^{m}{∥o_{ij}-\hat{o}\left(A,D,R_{i},t_{i},O_{j}\right)∥}^{2}$$

where $o_{ij}$ are the coordinates of feature j in imagei and $\hat{o}\left(A,D,R_{i},t_{i},O_{j}\right)$ is the projection of the corresponding 3D point $O_{j}$ with the distortion coefficients D, camera matrix A and the rotation matrix $R_{i}$ and translation vector $t_{i}$ as the relation between the camera and world coordinate system.

**Fig. 3:**
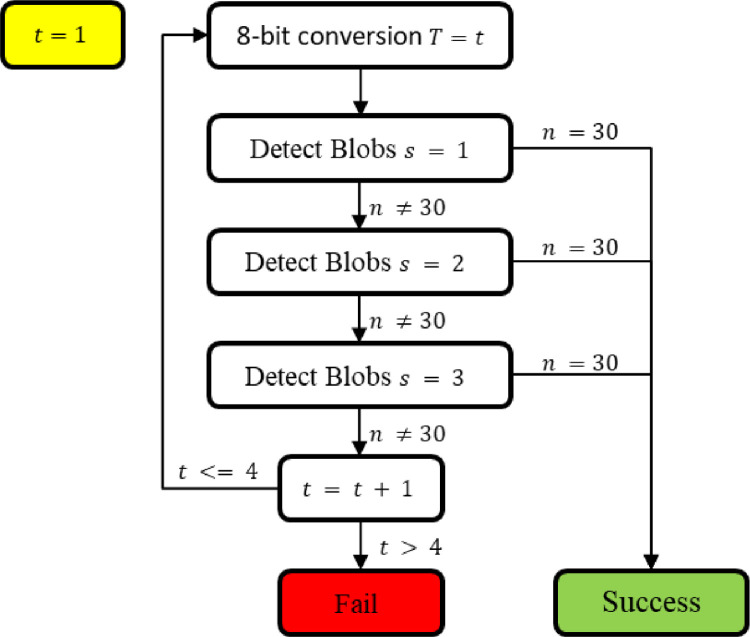
Flow chart of blob detection process for one image. T stands for different temperature ranges, s is the sensitivity of the blob detector and n is the number of detected blobs.

The process of detecting the blobs in an image is visualised in Fig. 3. The image was initially scaled to its temperature range (T, consisting of the minimum and maximum temperature found, and converted to 8 bit to make the blobs more visible. The aforementioned blob detector was configured with a sensitivity s and applied to the image. If the total number of blobs was found, the blob detection deemed successful, otherwise the *SimpleBlobDetector* reconfigured and applied again. In the case that three different sensitivities do not yield a successful result, the image was rescaled decreasing the temperature range by 5 degrees and repeating the blob detection. Whereas, if after 4 different temperature ranges, each of which has been analysed with the 3 sensitivity settings, the required number of blobs is not found, then the image is deemed a failure.

**Fig. 4:**
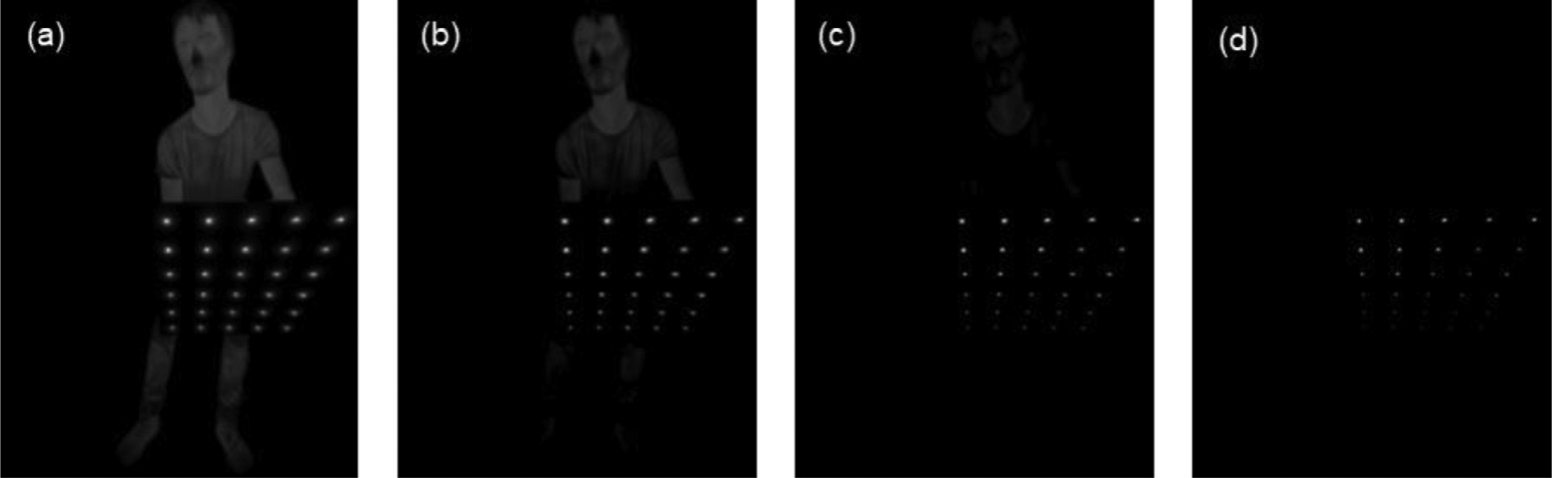
The effect of different temperature ranges of the 8-bit conversion of the image. (a) T = [20°C, 60°C], (b) T = [25°C, 60°C], (c) T = [30°C, 60°C], (d) T = [35°C, 60°C].

**Fig. 5:**
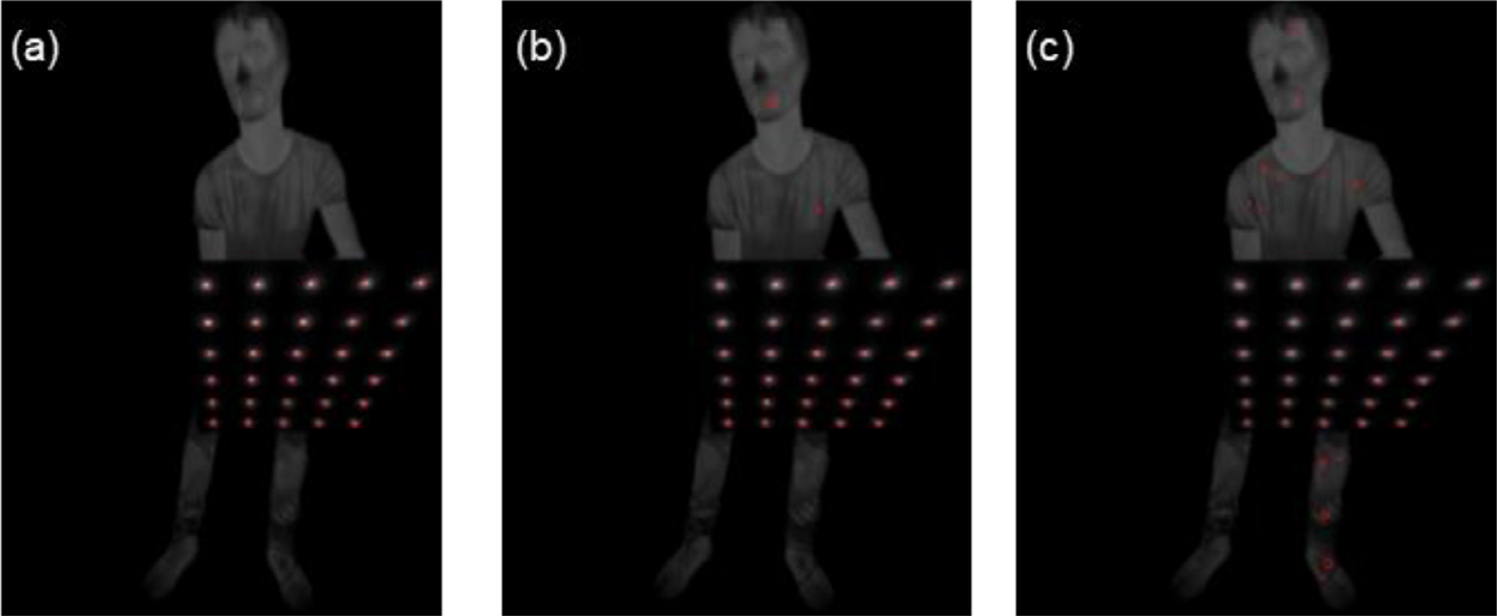
The effect of the three different sensitivity settings on the same image scaled to the temperature range *T = [20°C, 60°C].* (a) sensitivity s = 1, (b) sensitivity s= 2, (c) sensitivity s = 3.

This process compensates for the rather wide opening angle of the lens, which leads to a big variety of pattern positions to cover the whole frame as well as recording the pattern at an angle and the desired focus point. For example, enhanced blob size is perpendicular to image plane, while decreased blob size can depict high incident angle. The sensitivity parameters that have been utilised in this work, for the specific thermal camera and calibration pattern, are listed in Table 1. The different temperature ranged as well as the effect of different sensitivity settings was visualised in Fig. 4 and Fig. 5, respectively.

**Table 1:** Sensitivity parameters used for SimpleBlobDetector.

Sensitivity	*minThreshold*	*maxThreshold*	*thresholdStep*	*minArea*	*maxArea*
1	20	220	15	1	60
2	30	220	15	3	80
3	50	220	20	5	100

### Extrinsic calibration

To get a 3D point cloud and a corresponding thermal image from the 2D LiDAR sensor and thermal camera, were mounted on the linear conveyor as shown in Fig. 1b. The conveyor is moving the sensor setup with a constant velocity perpendicular to the LiDAR scanning plane, thus yielding a 3D point cloud. Similar to (Eq. (2)), the relation of a point in LiDAR coordinates $L={\left(X_{L},Y_{L},Z_{L}\right)}^{T}$ and the corresponding image point o = ${\left(x,y\right)}^{T}$ defined by

(6) $$\left(\begin{array}{c} x\\ y\\ 1 \end{array}\right)=A\left[\begin{array}{cccc} 1 & 0 & 0 & 0\\ 0 & 1 & 0 & 0\\ 0 & 0 & 1 & 0 \end{array}\right]\left[\begin{array}{cc} R & t\\ 000 & 0 \end{array}\right]\left(\begin{array}{c} X_{L}\\ Y_{L}\\ Z_{L}\\ 1 \end{array}\right),$$

with A being the camera matrix from (Eq. (3)), R the rotation and t the translation between the coordinate system of the thermal camera and LiDAR laser scanner. The latter variables are known as the extrinsic parameters, determined based on the relation of 2D image features (o) to their corresponding 3D LiDAR coordinates (*L*), which referred as feature pairs. For such set of point cloud and thermal image with m distinct feature pairs ${\left(o_{i},L_{i}\right)}_{i=1}^{m}$, rotationR and translation t between camera and LiDAR coordinate system are obtained by minimising

(7) $$\sum_{i=1}^{m}{∥o_{j}-\hat{o}\left(A,D,R,t,L_{j}\right)∥}^{2},$$

with $o_{i}$ as the pixel coordinates of blob i, $L_{j}$ as the corresponding 3D point in LiDAR coordinates, A and D as camera matrix and the distortion coefficients described in Eq. (3), ((4)) and $\hat{o}\left(A,D,R,t,L_{j}\right)$ as the projection of $L_{j}$ onto the image plane.

**Fig. 6:**
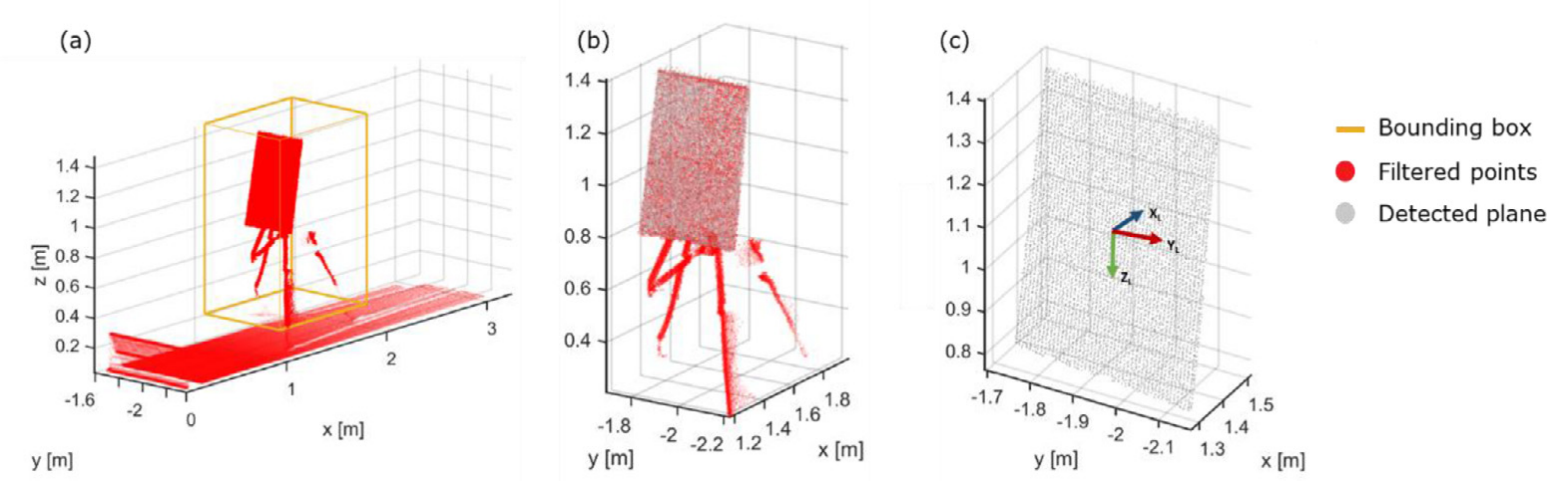
Processing of point cloud: (a) box crop to remove walls, floor and ceiling, (b) plane detection, (c) downsampled data with local coordinate system.

To extract the 3D LiDAR coordinates (L) of the features from the point cloud, a processing pipeline was applied (Fig. 6). More specifically, the point cloud was cropped with an axis aligned bounding box to remove ceiling, floor, and walls (Fig. 6Error: Reference source not found, a). This was done to ensure, that the biggest remaining plane in the point cloud is the calibration pattern itself. Moreover, the Open3Ds plane detection was applied to detect the prominent plane in the point cloud, using the random sample consensus algorithm (RANSAC) [36] (Fig. 6, b, white points), and all remaining points were removed (Fig. 6, b, red points). For the point cloud of the prominent plane, the bounding box was calculated, and the dimensions were checked to ensure, that they match with the size of the actual calibration pattern and all filtered points were removed.

A principal component analysis on the voxel downsampled *data* (Fig. 4, c) was performed to calculate the eigenvectors and corresponding eigenvalues. The eigenvectors and the centroid of the point cloud provided an initial estimation of the orientation and position of the calibration pattern ($T_{initial}$ and its local coordinate system relative to the laser scanner (Fig. 4, c). To optimise this calculation, a point cloud of the calibration pattern was synthetically sampled to serve as a perfect *model*, was roughly aligned with the subsampled point cloud using $T_{initial}$. Registering the *model* to the *data* using the iterative closest point (ICP) algorithm yields a correction transformation $T_{corr}$. The concatenation of $T_{initial}$ and $T_{corr}$ from the ICP algorithm provided the final transformation $T_{final}$ of the pattern relative to the scanner (Eq. (8))

(8) $$T_{final}=\left[\begin{array}{cc} R & t\\ 000 & 0 \end{array}\right]=T_{initial}*T_{corr}$$

**Fig. 7:**
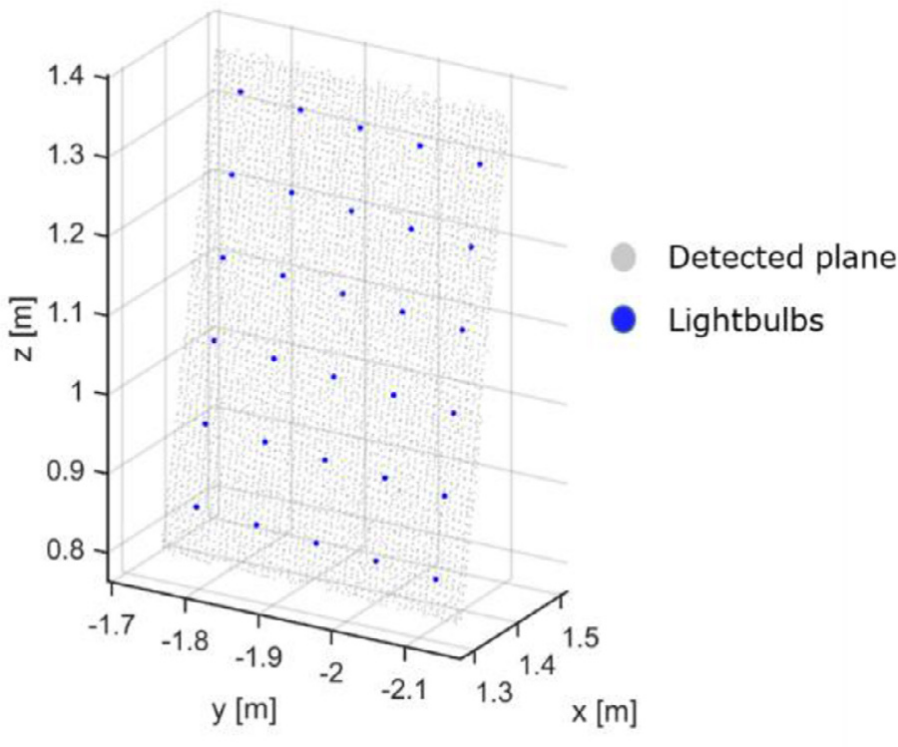
Lightbulb position determination.

When $T_{final}$ was specified based on the geometry of the calibration pattern (cf. Fig. 2), the position of the lightbulbs in LiDAR coordinates (L) was determined (Fig. 7). Since the calibration pattern is fixed relative to both sensors, Eq. (7) gives the desired orientation and translation between both sensors.

For the extrinsic calibration, a dataset consisting of 21 scans for different calibration pattern positions within the scanning area was recorded. Of those 21 scans, 6 could not be processed properly with all 6 scans having a section of the calibration pattern cut off due to bad placement within the scanning area. For each valid scan, the extrinsic parameters were determined according to the aforementioned pipeline (Fig. 3). Each set of parameters was then used to calculate the RMSE of the reprojection of all valid datasets according to

(9) $$rmse=\sqrt{\frac{\sum_{i=1}^{N}\sum_{j=1}^{M}∥{m\prime }_{i,j}-m_{i,j}^{2}∥}{N*M}},$$

with N being the number of valid scans, M being the number of points per scan, $m\prime_{i,j}$ being the jth point of scan i projected onto the image plane and $m_{i,j}$ being the jth point of scan i of the calibration pattern.

### Data Fusion

When all intrinsic and extrinsic parameters determined, temperature values from the thermal image were assigned to the corresponding 3D points. All points in the point cloud were projected in the image plane (Fig. 7). If the projected point lied within the image plane, the corresponding temperature value was assigned. Whereas, if the point was outside the plane, a value outside the temperature range of the dataset, in this case -10 °C, was assigned. This ensured that points outside of the field of view of the thermal camera were not omitted.

### Segmenting apple temperature

After calibration, the phenotype system was mounted on the circular conveyor in order to scan the fruit trees from both sides. The temperature values assigned in the corresponding 3D point cloud were based on the extrinsic calibration. According to Tsoulias et al., [27] rigid translations and rotations were applied on each point of the 3D point cloud, while alignment of pairing tree sides was carried out using ICP. The bivariate point density histogram was proposed to estimate the stem position of each tree (n = 20), while a cylindrical boundary was projected around the estimated stem positions to segment each individual tree.

For defining the position and shape of apples, the geometric feature of curvature (C) was calculated for each point of each 3D tree point cloud using the k-nearest neighbors algorithm [28]. For this purpose, the local neighborhood of points $P_{i}$= [$x_{i},\;y_{i},\;z_{i}$] was analysed in 3D coordinates. The total number (N) of P_i_ within each tree's cloud was used to estimate the mean of all nearest neighbors

(10) $$\tilde{P}=\frac{1}{N}\sum_{i=1}^{N}\left(P_{i}\right)$$

After mean centering, each $P_{i}$ with $\tilde{P}$ value per nearest neighbor's set and decomposition of covariance matrix. The latter was decomposed based on the singular value decomposition, producing the eigenvalues (λ_1_, λ_2_, λ_3_), which were classified according to decreasing percentage of explained variance in the data. The eigenvalues were scaled between 0 and 100, allowing the comparison of different clusters. More specifically, the values closer to 100, the higher the likelihood for shape of points to be curved. The probability density function was performed to define the thresholds of curvature (C_th_) and LiDAR's backscattered reflectance (R_th_) defining the range of apple points in terms of C and backscattered reflectance (C_A_ and R_A_). The points that fulfilled the criteria of C_th_ ≤ C_A_, and R_th_ ≤ R_A_ were segmented and categorised as apples. This allowed to define the temperature values on the surface of apples by means of LiDAR (FST_LiDAR_). The temperature on fruit surface was manually measured (FST_Manual_) (n = 285) with an infrared thermometer (Microscanner D501, Exergen, Watertown, USA) and compared with the correspondent averaged FST_LiDAR_. The detected apples were categorised, in west and east, based on their position on the tree side.

### Evaluation

**Fig. 8:**
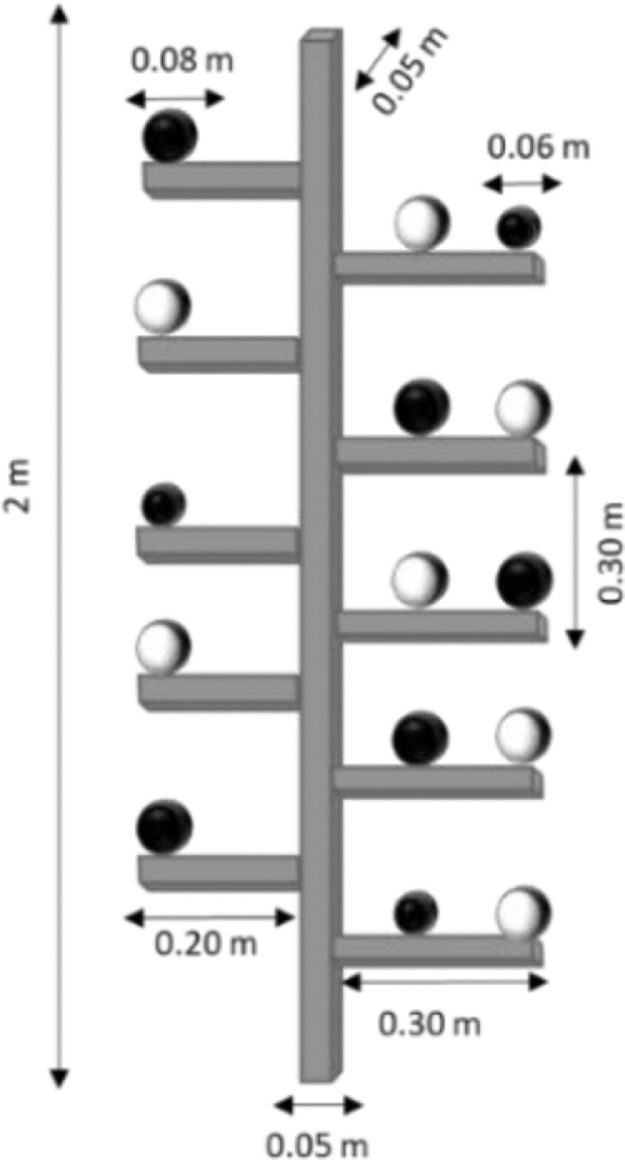
Representation of the metal tree frame of known distances with sphere targets.

A metal tree frame with dimensions 2 m × 0.30 m × 0.05 m was constructed to assess the measuring uncertainty of the phenotypic system in terms of temperature (Fig. 8). Five bars with 0.30 m distance from each other were placed horizontally on each side of a metal trunk. Sphere targets of 60 mm (n = 3) and 80 mm (n = 12) size were applied to assess the temperature derived by the phenotypic platform. The spheres were coated with white barium sulphate (BaSO_4_, CAS Number: 7727-43-7, Merck, Germany) and blackened urethane (S black, Avian Technologies, New London, NH, USA) for acquiring the minimum (S_W_) and maximum (S_B_) T_LiDAR_ on the sphere surface. The phenotypic system was utilised to scan the metal frame indoors and outdoors in the orchard, and an infrared thermometer to manually acquire the temperature on the sphere surface (T_Manual_).

Descriptive statistics were applied to all datasets capturing minimum (min), maximum (max), mean, standard deviation (SD). A regression analysis was performed to quantify linear relationships between the manual measurements and the detected temperature by means of LiDAR, and RMSE, mean bias error (MBE). Descriptive statistics were carried out by Matlab (v.R2018b, Mathworks Inc., Natick, MA, USA).

## Results

### Intrinsic calibration

**Fig. 9:**
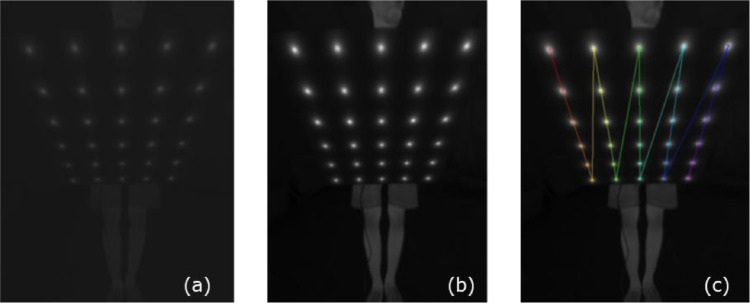
(a): Raw Thermal Image, (b): Scaled to Temperature Range, (c): Detected and Sorted Blobs.

Sample images for the intrinsic calibration process are visible in Fig. 9. The raw thermal image (left) is scaled to a temperature range (middle) and the blobs are detected and sorted (right) according to the visible color scheme from red to violet. For our dataset of 140 images, 10 images were discarded, with all 10 having at least one of the blobs missing due to poor alignment of the pattern while recording the images. The root mean squared error (RMSE) of the reprojection yielded 0.33.

### Extrinsic calibration

**Fig. 10:**
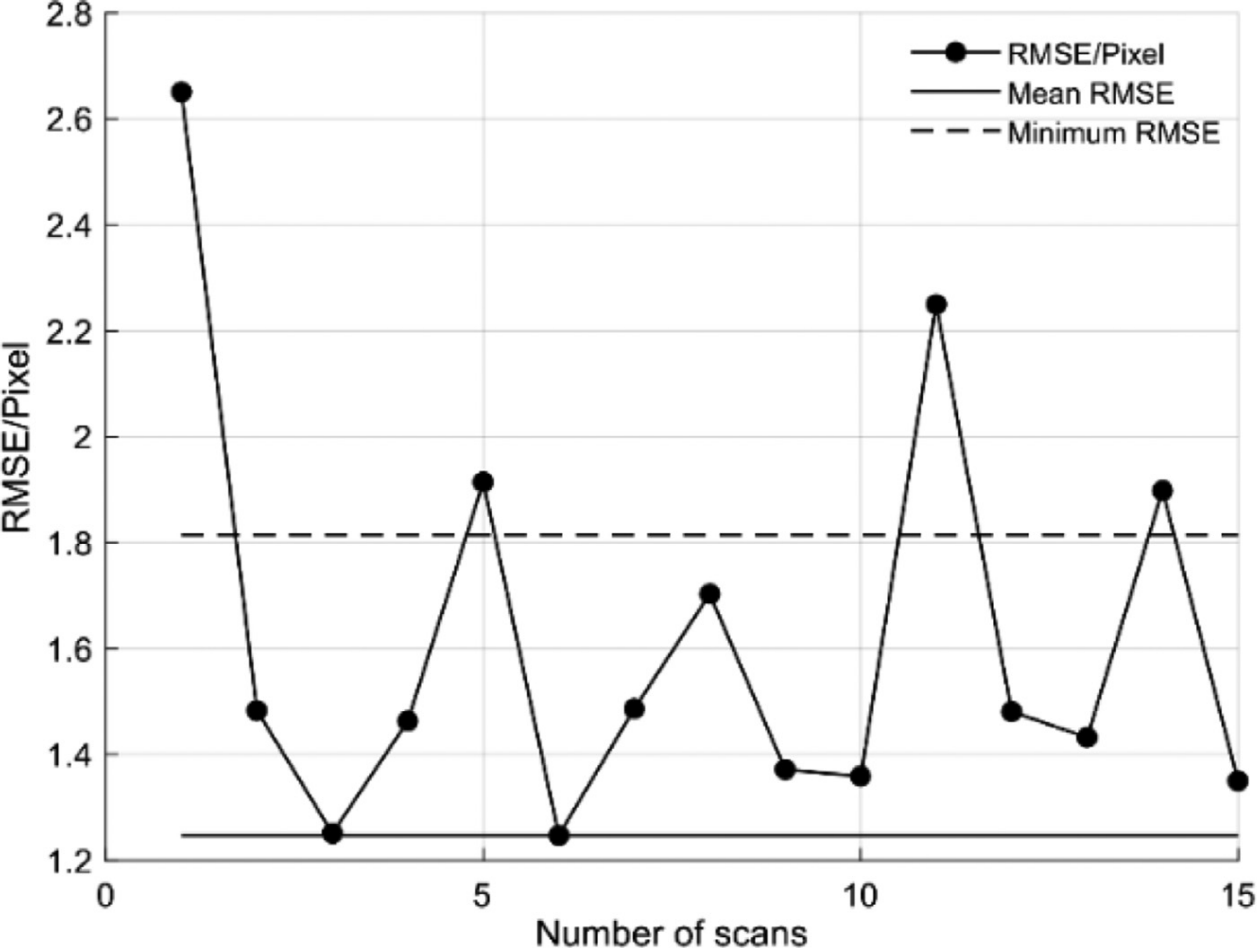
Root Mean Square Error (RMSE) of Extrinsic Calibration per Scan. The RMSE yielded by averaging all parameters elementwise is shown as a line in grey, the Minimum RMSE is shown as a line in yellow.

The calculated RMSE was shown in Fig. 10. Calculating the mean of all extrinsic parameters element-wise yielded an RMSE of 1.82 (grey line), thus it was decided to further use the parameter set with the lowest RMSE (Nr. 6, 1.25).

### Data fusion

**Fig. 11:**
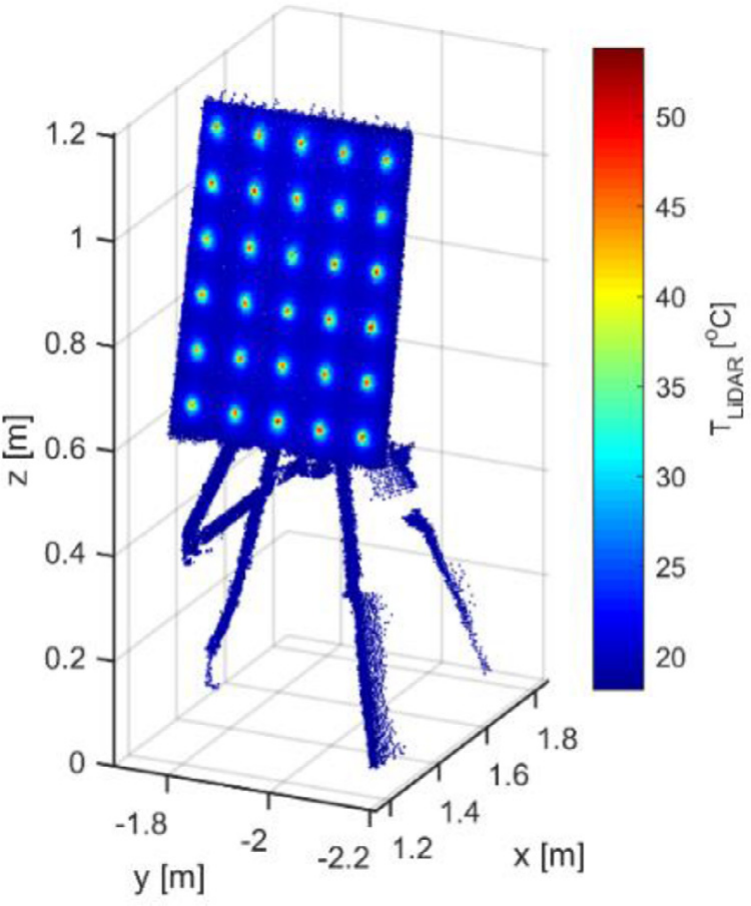
Point cloud of the calibration pattern.

An example of a point cloud of the calibration pattern, containing LiDAR and temperature information was depicted in Fig. 8. Temperature values were scaled from 15 to 55 °C. The center of blobs showed a mean T_LiDAR_ of 46.3 °C with an SD 2.93 °C, while a less pronounced mean T_LiDAR_ of 21.41 °C the 3.03 °C was found in the rest points of the calibration pattern Fig. 11.

### Evaluation

**Fig. 12:**
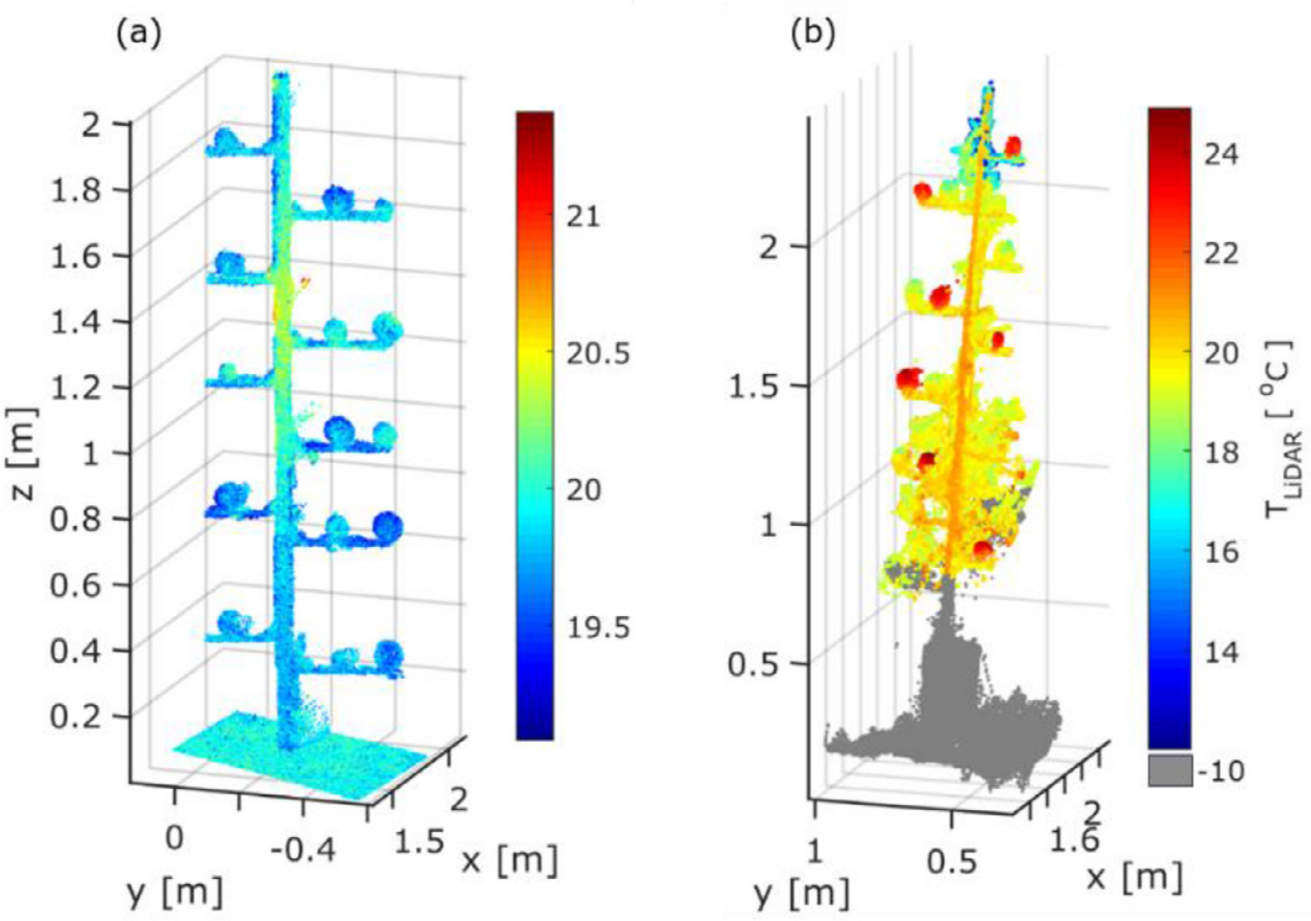
The resulting thermal point cloud of the (a) metal tree indoors and (b) outdoors.

The values of T_LiDAR_ on the white (S_w_) and black (S_B_) surfaces appeared above 19.54 °C and below 19.84, respectively (Table 2). The temperature difference between the spheres was marginally differed, since no passive heat was applied, and the ambient temperature of the room remained at 19 °C. The T_LiDAR_ was related to the T_Manual_, revealing an adjacent coefficient of determination (R^2^_adj_) of 0.95 RMSE = 0.02 °C in S_B_ and 0.94 with and 0.01 °C in S_W_. Generally, high measuring uncertainty was noticed on spheres, when the metal construction placed in field conditions (Fig. 12 b), particularly in the black coated spheres. The minimum and maximum T_LiDAR_ showed 1.5 °C difference on the surface of S_B_.

**Table 2:** Results of LiDAR detected temperature (T_LiDAR_) of spheres on the metal tree indoors and outdoors (n = 15), regarding maximum (max) [°C], minimum (min) [°C], standard deviation (SD) [°C], mean bias error (MBE) [°C], root mean square error (RMSE) [°C], and adjusted coefficient of determination (R^2^_adj_).

		min	max	mean [°C]	SD	MBE	RMSE	R^2^_adj_
Indoors	S_B_	19.72	19.84	19.69	0.08	0.02	0.02	0.95
	S_W_	19.54	19.82	19.84	0.07	0.01	0.01	0.94
Outdoors	S_B_	20.81	22.31	22.20	0.67	-0.03	0.11	0.97
	S_W_	19.18	19.63	19.39	0.18	0.01	0.04	0.95

**Fig. 13:**
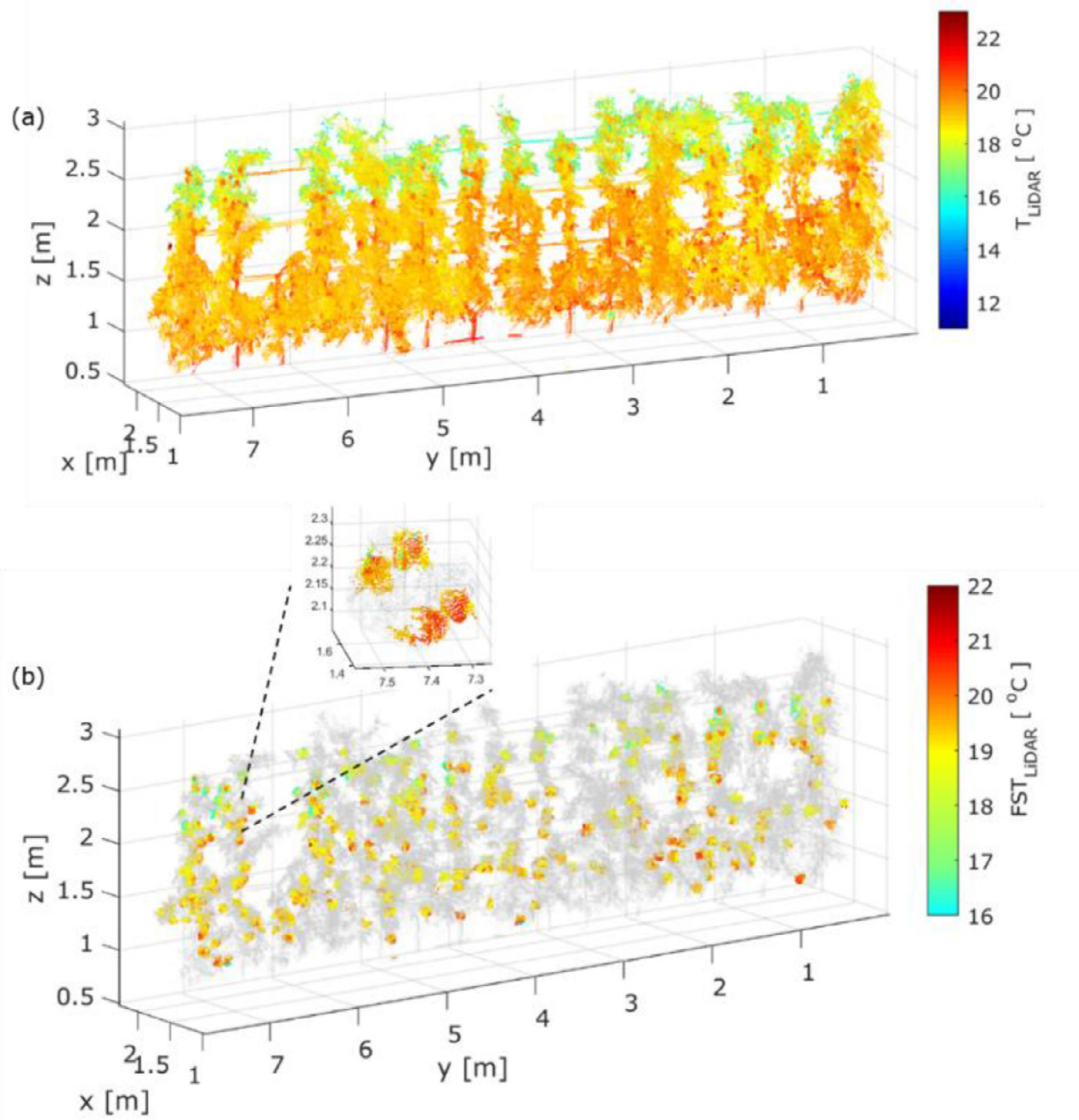
Representation of (a) 3D thermal point cloud and (b) segmented temperature on fruit surface (FST_LiDAR_) [°C] in sampled trees measured with at DAFB_120_.

The methodology was applied in the orchard with a total number of 285 apples, 130 days after full bloom. The temperature varied in the 3D point cloud of the trees (Fig. 13a). Tree organs, found above 2 m, revealed reduced T_LiDAR_ not exceeding 18.2 °C. Moreover, the T_LiDAR_ on stem points showed a mean value of 20.6 °C with 0.65 °C standard deviation. After the application of fruit detection algorithm, the shape from 272 with an 89.7% F1 score was detected. The FST_LiDAR_ ranged between 16 and 22 °C (Fig. 13b).

**Fig. 14:**
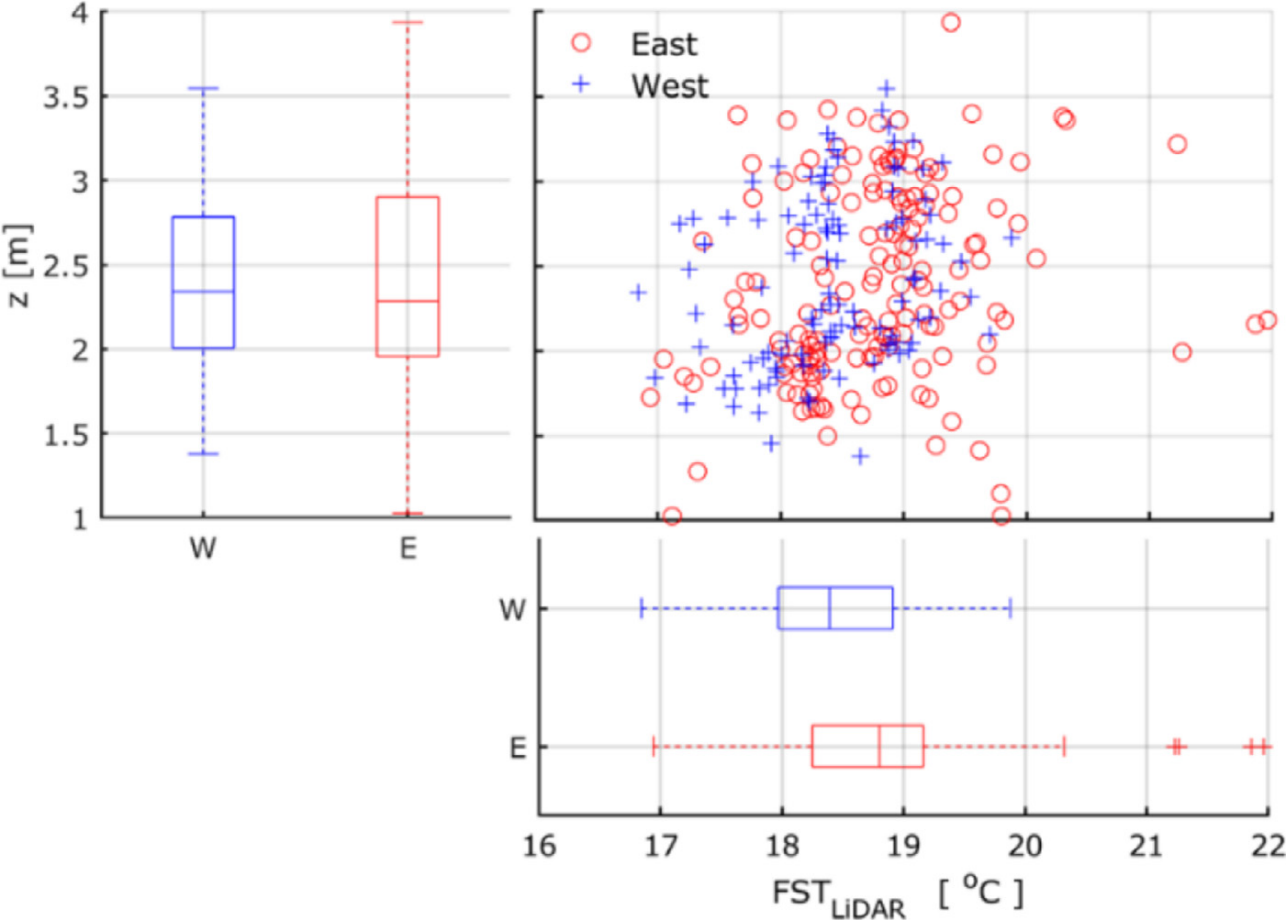
Scatter plot and marginal box-Whisker plot of the segmented temperature on fruit surface (FST_LiDAR_) and of fruit height, categorised based on the west (W) and east side (E) of the tree. The standard deviation is represented by lower and upper edges of the box, the dash in each box indicates the average.

The FST_Manual_ was related with FST_LiDAR_ of apples in the west and east side of the trees, resulting in an R^2^_adj_ of 0.91 and 0.99 with an RMSE of 0.25 and 0.01, respectively. The fruit located in the east side of tree developed an enhanced average FST_LiDAR_ (18.8 ± 0.75 °C), while a less pronounced value (18.3 ± 0.61 °C) was observed in the west side (Fig. 14). In parallel, apples from both sides depicted a similar range in terms of height.

Overall, the monitoring system demonstrated great potential for monitoring FSTLiDAR in apples. Moreover, the enhanced field of view of LiDAR laser scanner can determine the FST, which derive from the entire 3D tree profile, allowing to model fruit temperature and improve decision making in the orchard. The frequent acquisition of FSTLiDAR can be utilised as control measures to detect damaged fruit on the tree, increasing fruit storability and reducing food waste. The acquired FST information could be utilized, in future, for predicting various abiotic stresses (e.g. sunburn) and comprehending its effect on soluble solid content in relation with the position of the fruit in the tree canopy. The described methodology with specific customization, based on sensor availability, could be utilised for heat-stress monitoring in other perennial specialty crops.

## Conclusions

The developed methodology was able to register the thermal images on LiDAR 3D point cloud with the lowest RMSE of 1.2 MSE/pixel. Application of the metal construction allowed the evaluation of the extrinsic calibration, presenting a highest 0.02 °C RMSE with 0.95 R^2^_adj_ in the lab, and 0.11 °C RMSE with 0.97 under field conditions. It also provided meaningful information about the FST_LiDAR_ on apples, which correlated strongly with the FST_Manual_ (R^2^_adj_ = 0.99) in the east side of the tree. The values of apples in the east side of tree showed enhanced FST_LiDAR_ values compared to the west side. In summary, the phenotypic system was able to detect the temperature on apple surface, a result that can be utilised in the monitoring and prevention of fruit sunburn.

## Declaration of Competing Interest

The authors declare no conflict of interest.

## Appendix Supplementary materials

Image, application 1
